# Cystic Duct Diameter as a Key Predictor for Closure Difficulties in Laparoscopic Cholecystectomy

**DOI:** 10.7759/cureus.87254

**Published:** 2025-07-03

**Authors:** Shunsuke Tabe, Norikazu Yogi, Ayu Kato, Sojun Hoshimoto, Yoshifumi Ikeda, Masayuki Ohtsuka, Masaru Miyazaki

**Affiliations:** 1 Digestive Disease Center, International University of Health and Welfare, Mita Hospital, Tokyo, JPN; 2 General Surgery, Chiba University Graduate School of Medicine, Chiba, JPN

**Keywords:** cystic duct, endoscopic retrograde cholangiography, gallbladder stones, laparoscopic cholecystectomy, magnetic resonance cholangiopancreatography

## Abstract

Background

Although a 5 mm diameter metal clip is commonly employed for cystic duct (CD) closure, it may sometimes be inadequate due to CD dilation. Various widely used preoperative scoring systems can predict the difficulty of intraoperative manipulations, but these systems do not mention CD closure methods. In this study, we identified several preoperative factors related to these instances.

Methodology

We selected 192 patients who underwent laparoscopic cholecystectomy at our institute. The standard group comprised cases of CD closure with a 5 mm metal clip, while the unusual group included cases of CD closure involving 10 mm or larger clips, suturing, ligation, or a laparoscopic stapler. The CD diameter was measured using magnetic resonance cholangiopancreatography (MRCP) imaging.

Results

In total, 20 (13%) cases of gallbladder stones were treated via unusual methods. A univariate analysis showed that the occurrence of common bile duct (CBD) stones and the frequency of use of endoscopic retrograde cholangiography were higher in the unusual group compared with the standard group, and CBD and CD diameter values were greater in the unusual group. Moreover, CD anatomical variations were also associated with the use of unusual methods for CD closure. The cutoff values for CD and CBD diameters were 4.22 mm and 6.25 mm, respectively. A multivariate analysis indicated that CD dilation (>4.22 mm) was strongly associated with difficulties in CD closure.

Conclusions

If CD dilation is detected via preoperative MRCP imaging, the surgeon should carefully consider the type of CD closure method to be employed.

## Introduction

Laparoscopic cholecystectomy (LC) is widely performed for patients who have gallbladder stones (GBS) or gallbladder polyps, and other related conditions. Recently, 93.0% of cholecystectomies have been performed laparoscopically [1]. To ensure safety during LC, it is generally recommended to perform a critical view of safety and identify both the gallbladder artery (GBA) and the cystic duct (CD) [2]. However, the level of difficulty of this operation may vary due to factors such as inflammation from cholecystitis and the positioning of the GBA [3]. Consequently, these structures may not be easily detectable, which can compromise safety. To prevent intraoperative complications such as bile duct injury, hemorrhage, or bile leakage after surgery, various scoring systems have been utilized [4-7]; however, these systems cannot predict the level of difficulty for CD closure.

It is crucial to close the CD and completely prevent bile leakage after LC. A metal or polymer clip with a shaft diameter of 5 mm (5 mm clip) is generally used to close the CD [8]. Therefore, only working trocars measuring 5 mm are necessary, except for the camera trocar. Reduced-port surgeries, such as single-incision laparoscopic cholecystectomy or three-port cholecystectomy [9-11], are often performed in patients without severe inflammation. Occasionally, a dilated CD that cannot be ligated with a 5 mm clip occurs unexpectedly without inflammation. In such cases, it is necessary to switch from the 5 mm clip to a larger one (10 mm clip), which cannot be inserted through the 5 mm working trocar. In severe cases, a laparoscopic stapler (LS) is required to close the dilated CD [12,13]. This poses a slight obstacle to performing the operation smoothly.

The preoperative scoring systems mentioned above predict the level of difficulty in carrying out LC and involve parameters such as operating time, hemorrhage, and postoperative complications [4-7], but do not include the method used for CD closure. Although a few studies have mentioned a correlation between the anatomical variations of the CD and GBS or CD size and biliary events [14], the difficulty of LC in terms of the methods employed for CD closure has not been adequately considered. Some studies have shown that an inflamed or dilated CD may preclude the use of standard metal or polymer clips due to incomplete CD traversal [15]. LS presents one alternative for complete CD ligation; however, the complication rate in cases of LS use is higher than expected [15]. There is still controversy regarding which devices are suitable for closing an enlarged CD.

Here, we retrospectively analyzed patients who underwent LC at our institute to identify preoperative factors that predict the difficulty of CD closure. Our present findings may help prevent intraoperative complications and assist young surgeons in selecting the appropriate CD closure methods for LC.

## Materials and methods

Study population and data collection

The patients who underwent LC between 2020 and 2022 at the International Welfare and Health University, Mita Hospital, Japan, formed the patient cohort of this study. The method used to close the CD was obtained by accessing the surgical notes from the records of our institution electronically. Individual patient data were collected as follows: age, sex, body mass index (BMI), the existence of cholecystitis at diagnosis, the existence of GBS and common bile duct stones (CBDS), preoperative endoscopic retrograde cholangiography (ERCP), the diameter of the CD and common bile duct (CBD), anatomical variations of CD type [14], admission type for operation, intraoperative information such as size of epigastric trocar, operative time, amount of blood loss, and the total days of hospital stay. This study was approved by the Institutional Review Board of Mita Hospital (approval number: 24-MT-007).

Definitions

The exclusion criteria for our patient cohort were as follows: (1) those who had not undergone magnetic resonance cholangiopancreatography (MRCP); (2) those with a suspicion of gallbladder cancer; (3) those diagnosed with Mirizzi syndrome; and (4) those with a left-sided gallbladder. We divided the patients into two groups. The standard group was defined as patients whose CDs were closed using a 5 mm clip (either metal or polymer), which corresponds to the size of the device shaft. In contrast, the unusual group was defined as patients whose CDs were closed using a 10 mm clip, ligature device (endo-loop), laparoscopic suturing, or an LS. This information was obtained from the electronic operative notes of our institution.

Evaluation of the images

**Figure 1:**
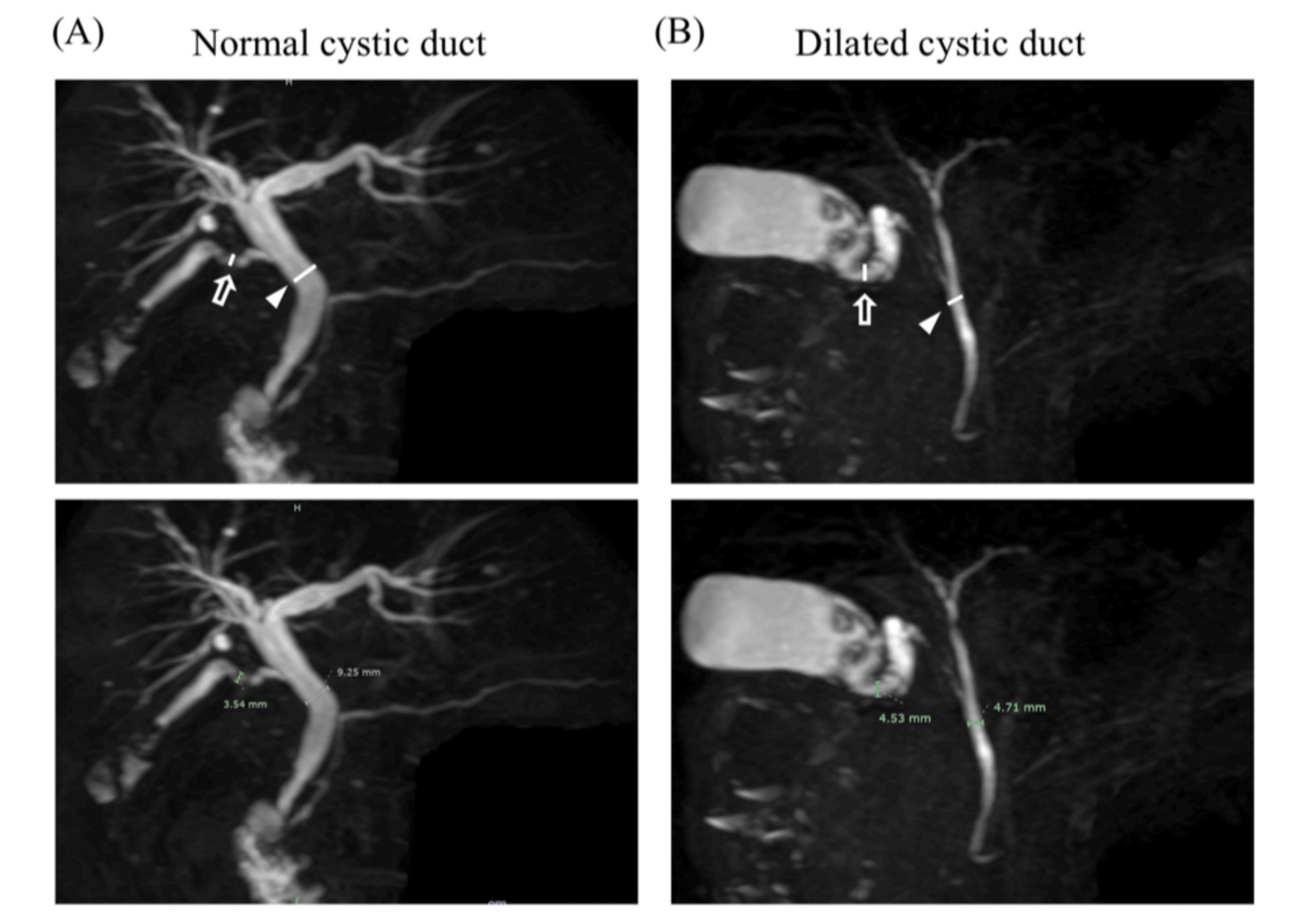
Magnetic resonance cholangiopancreatography (MRCP) images. (A) The diameters of the cystic duct were represented in both two- and three-dimensional construction images via MRCP. (B) Intraoperative images from laparoscopic cholecystectomy of patients with the MRCP image above.

**Figure 2:**
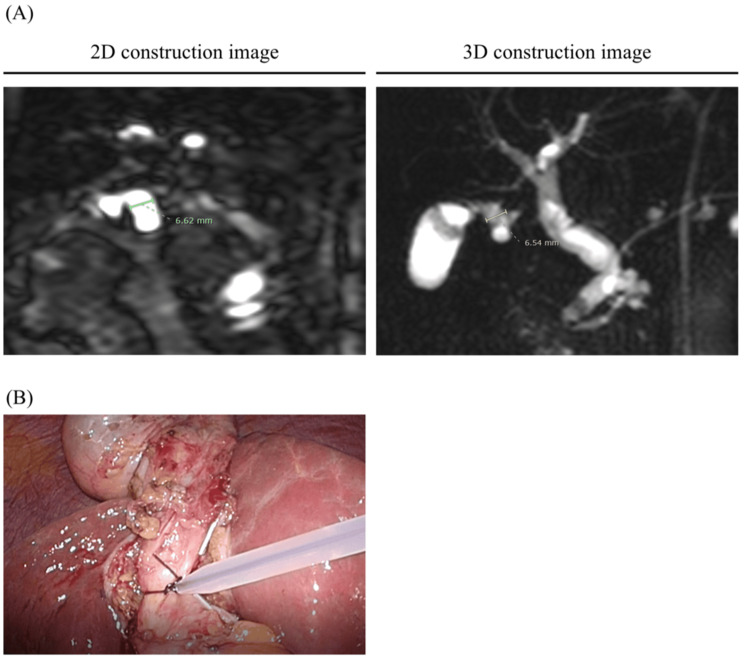
Magnetic resonance cholangiopancreatography (MRCP_ images. (A) The diameters of the cystic duct were represented in both two- and three-dimensional construction images via MRCP. (B) Intraoperative images from laparoscopic cholecystectomy of patients with the MRCP image above.

MRCP imaging was performed on patients after an overnight fast using 3.0 T MRI scanners (Achieva 3.0T TX, Philips, Amsterdam, Netherlands). The diameter of the CD and CBD was measured using MRCP imaging and employing 2D or 3D imaging to assess the clarity of the construction. The length of the CD was detected from just below the Hartmann’s pouch, and the CBD was measured after connecting it (Figures 1, 2). The anatomical variation of the CD was defined via 3D construction from MRCP imaging as previously reported [14]. If the 3D image was unclear, we used the 2D image instead. Different surgeons in our department measured these sizes.

Statical analysis

Statistical analyses were conducted using JMP Pro software version 18.0 (JMP Statistical Discovery, North Carolina, USA). Baseline patient characteristics were compared using independent chi-square tests for categorical variables and analysis of variance for continuous variables. Univariate logistic regression was employed in the preliminary analysis to evaluate factors influencing the method of CD closure. Multivariate logistic regression was subsequently conducted to further analyze significant variables identified in the univariate analysis. Odds ratios (ORs) and 95% confidence intervals (CIs) were reported. Receiver operating characteristic (ROC) curve analysis was applied to determine optimal cutoff values for CD and CBD diameters, maximizing sensitivity and specificity for classifying patients based on the CD closure method. Data are presented as mean ± standard deviation (SD), and statistical significance was set at p-values <0.05.

## Results

**Figure 3:**
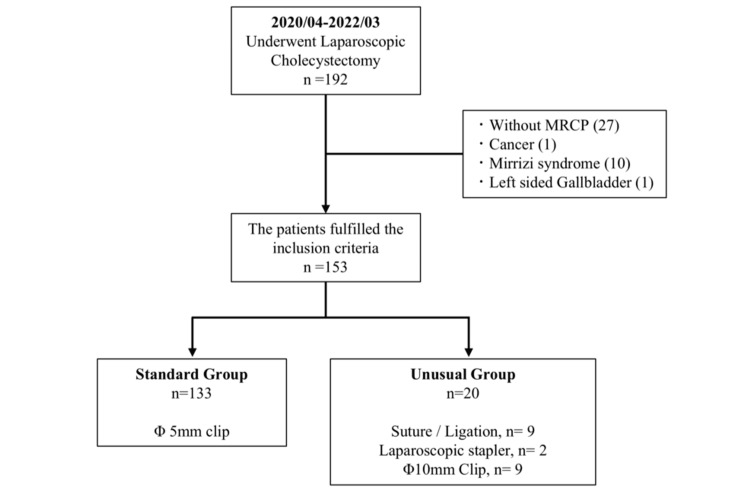
Flowchart showing the cohort selection. MRCP: magnetic resonance cholangiopancreatography

Between 2020 and 2022, 192 patients underwent the LC procedure at our institute. In total, 39 patients were excluded based on the exclusion criteria. Overall, 27 patients did not undergo MRCP imaging, 10 patients were diagnosed with Mirizzi syndrome, and one patient was diagnosed with gallbladder cancer and a left-sided gallbladder. Finally, 153 patients were divided into the following two groups: 133 (86.9%) patients underwent LC using the standard method, while the other group consisted of 20 (13%) patients, where unusual methods of CD closure were used (Figure 3). Among the 20 patients in the unusual methods group, nine underwent LC using suturing or a ligature device (Endo loop), nine were treated with a 10 mm clip, and two were treated with LS.

Patient characteristics

Table 1 shows a comparison between the two patient groups using univariate analysis. There was no significant difference in age, sex, BMI, and, surprisingly, the presence of cholecystitis, which was diagnosed before the surgery, as well as the occurrence of GBS (p = 0.86 and p = 0.89, respectively). Emergency operation status did not impact the method used for CD closure (p = 0.97). The unusual group was diagnosed with CBDS more often than the standard group (25% vs. 6.7%; p < 0.01) and underwent ERCP at a higher rate (40% vs. 9.7%; p < 0.01).

**Table 1:** Characteristics of patients in the standard and unusual groups. BMI: body mass index; CBD: common bile duct; ERCP: endoscopic retrograde cholangiopancreatography

	Standard (n = 133)	Unusual (n = 20)	Chi-square value	F-value	P-value
Age	61.1 ± 13.4	56.2 ± 14.9	-	3.14	0.08
Male/Female	53/80	6/14	0.16	-	0.39
BMI	23.3 ± 4.0	24.5±3.3	-	0.62	0.89
Cholecystitis					
Yes(%)	31 (23.3)	5 (25)	0.08	-	0.86
No(%)	102 (76.6)	15 (80)			
Presence of gallbladder stones					
Yes (%)	95 (71.4)	14 (70)	0.03	-	0.89
No (%)	38 (28.5)	6 (30)			
Presence of CBD stone (%)					
Yes (%)	9 (6.7)	5 (25)	8.32	-	<0.01
No (%)	124 (93.2)	15 (75)			
Preoperative ERCP					
Yes (%)	13 (9.7)	8 (40)	12.4	-	<0.01
No (%)	120 (90.2)	12 (60)			
Diameter of CBD (mm)	5.44 ± 1.71	6.97 ± 2.18	-	9.34	<0.01
Diameter of cystic duct (mm)	2.49 ± 0.85	5.89 ± 1.58	-	229.6	<0.01
Emergency surgery					
Yes (%)	13 (9.7)	2 (10)	0.07	-	0.97
No (%)	120 (90.2)	18 (90)			
Variation of cystic duct (%)					
Type L	93 (92.0)	8 (7.9)	21.8	-	<0.01
Type N	38 (88.3)	5 (11.6)			
Type S	2 (22.2)	7 (77.7)			
Intraoperative characteristics					
Size of epigastric port 5 mm/others	106/27	8/12	2.78	-	<0.01
Amount of blood loss (mL)	11.8 ± 37.6	30.8 ± 67.4	-	3.45	0.03
Operating-time (minute)	100 ± 42.3	141.8 ± 54.3	-	10.5	<0.01
Hospital stay (days)	5.02 ± 4.41	4.15 ± 2.05	-	0.75	0.1936

The diameter values of CD and CBD acquired via MRCP imaging were compared in the two groups. The mean CD diameter in the unusual group was larger than that in the standard group (5.89 ± 1.58 mm vs. 2.49 ± 0.85 mm; p < 0.01). The mean CBD length was also significantly larger in the unusual group (6.97 ± 2.18 vs. 5.44 ± 1.71 mm; p < 0.01). This finding was associated with the higher occurrence of ERCP conducted in the unusual group. Moreover, the anatomical variations of the CD [14] were related to the closing method utilized. The occurrence of type S CD was higher in the unusual group (n = 7, 77.7% vs. n = 2, 22.2%), while the occurrence of type L CD was lower in the standard group (n = 8, 7.9% vs. n = 93, 92.0%). To verify how the CD closure method affects intraoperative manipulation, we analyzed the intraoperative characteristics of the two groups. The epigastric trocar selected for the unusual group was of a larger size (p < 0.01). The amount of blood loss and operating time was higher in the unusual group; however, the length of hospital stay did not differ between the two groups.

Factors associated with the need for unusual methods

**Figure 4:**
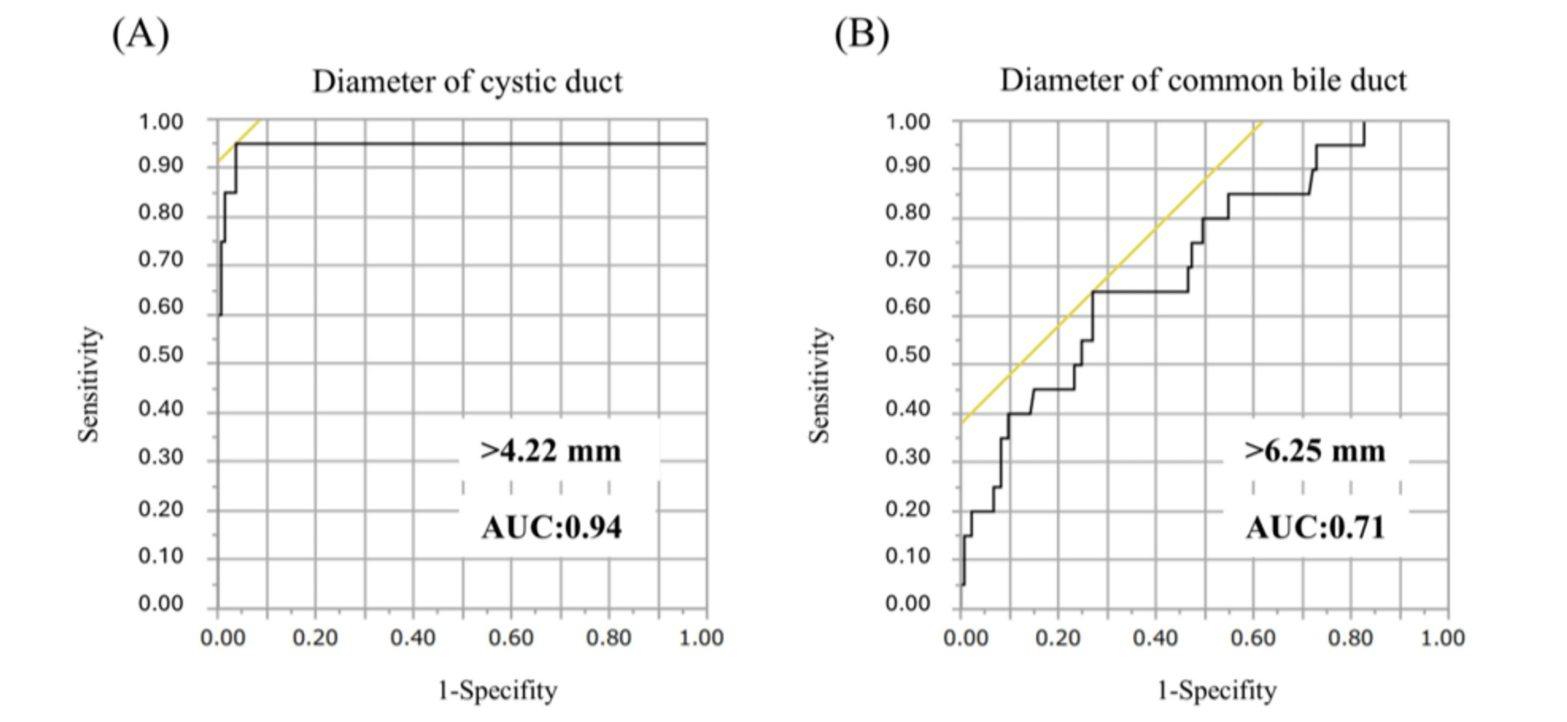
Receiver operating characteristic curves for the diameter of CD and CBD. (A, B) The cutoff value was 4.22 mm (AUC = 0.84, p < 0.001) for CD and 6.25 mm for CBD (AUC = 0.71, p < 0.01). AUC: area under the receiver operating characteristic curve; CBD: common bile duct; CD: cystic duct

The enlarged CD and CBD observed preoperatively via MRCP may increase the difficulty of CD closure during LC. We plotted the ROC curves for CD diameter and CBD length (Figure 4). The cutoff values for these parameters were 4.22 mm for CD (AUC = 0.94) and 6.25 mm for CBD (AUC = 0.71). Unusual methods of CD closure were needed if the CD diameter and CBD length exceeded these values.

A multivariate analysis (Table 2) also revealed that dilation of the CD (>4.22 mm) was the most significant factor affecting the difficulty of CD closure (p < 0.01). Conversely, preoperative cholecystitis was associated with a prolonged operative time and increased intraoperative blood loss; however, it did not influence the duration of hospital stay (Table 3).

**Table 2:** Multivariate analysis of risk factors for unusual methods. CBD: common bile duct; ERCP: endoscopic retrograde cholangiography; OR: odds ratio from univariate analysis; CI: confidence interval

	Odds ratio	95% CI	P-value
CBD stone (+)	7.64	0.28–201.7	0.21
Preoperative ERCP (+)	0.75	0.13–13.1	0.8
Type S cystic duct	18.8	0.5–708.8	0.06
Diameter of CBD >6.25 (mm)	1.02	0.07–13.2	0.99
Diameter of cystic duct >4.22 (mm)	299.6	22.4–4006.8	<0.001

**Table 3:** Univariate analysis for patients with and without cholecystitis. CBD: common bile duct

	Without cholecystitis (n = 117)	Cholecystitis (n = 36)	P-value
Diameter of cystic duct (mm)	2.9 ± 0.15	2.9 ± 0.25	0.9288
Diameter of CBD (mm)	5.5 ± 0.18	6.1 ± 0.32	0.1079
Standard/Unusual	102/15	31/5	0.8
Blood loss (mL)	11.4 ± 38.5	23 ± 54.1	0.135
Operation time (minute)	99.4 ± 41.8	125 ± 53.9	0.0032
Hospital stay (days)	4.1 ± 3.12	7.4 ± 5.91	0.0001

## Discussion

Since the first report of LC was published in Japan in 1990 [16], the strategy and techniques of the procedure have been well established, ensuring its safe execution. This is often regarded as the initial step in the surgical training of young surgeons and trainees. Therefore, careful attention must be paid to the intraoperative and postoperative complications associated with LC. Our findings suggest that surgeons should reconsider the method of CD closure when MRCP results indicate CD enlargement. Incomplete CD closure may lead to postoperative bile leakage and intra-abdominal abscess formation [15], requiring the surgeon to consider alternative techniques such as using an LS or larger clips. In recent years, LC has been performed by young surgeons as an introductory procedure at various institutions [17]. However, inflammation and adhesion around the gallbladder can increase procedural difficulty. In this study, preoperative MRCP imaging results were found to predict the difficulty of CD closure, and this information could help prevent unexpected intraoperative complications.

In general, acute or chronic cholecystitis could complicate intraoperative procedures. Several difficulty scoring systems have indicated that factors such as cholecystitis, cholangitis, preoperative ERCP findings, and gallbladder wall thickness contribute to the complexity of LC [4-7]. According to the 2018 Tokyo Guidelines, diffuse scarring in the Calot’s triangle area and around the gallbladder increases the difficulty of intraoperative procedures [2]. Although many scoring systems define the level of difficulty based on operative time, perioperative complications, and conversion to open abdominal surgery [4-7], there are few reports addressing the impact of the CD closure method. Our results indicate that the presence of acute cholecystitis does not affect CD closure; however, both blood loss and operative time were significantly increased (Table 3). Consistent with these findings, blood loss was significantly higher in the unusual group (Table 1). This can be attributed to the well-developed peri-bile duct vascularization [18], where CD dilation may lead to increased vascularity in the surrounding area. Even if no evidence of cholecystitis is found before surgery, surgeons should anticipate the possibility of oozing around the CD in cases of an enlarged CD.

We believe that in patients with a dilated CD detected before surgery, the gallstones may either pass through the CD or become impacted within it. Our findings indicate that anatomical variations of the CD may be one of the risk factors that necessitate the use of an unusual closure method (Table 2). Few studies have investigated the correlation between CD anatomical variations and the occurrence of GBS. Park et al. classified CD anatomical variations into types I to IV and reported that these differences contributed to the incidence of gallstones [14]. They noted that bile stasis and increased bile viscosity are more likely to occur in cases with a long CD, which subsequently contributes to the development of gallstones. Moreover, some reports suggest that a long CD may contribute to the development of secondary or residual stones after LC [19]. It remains controversial whether the residual CD length is a risk factor for the incidence of gallstones after LC, as further research is needed to determine the optimal dissection point for patients with a long CD.

We have defined the unusual CD closure methods to include the use of 10 mm clips or other techniques. The term “10 mm” refers to the diameter of the device shaft and is not indicative of the size or length of the clip itself. This distinction is important because 10 mm clips require a larger trocar (10-12 mm epigastric trocar) compared with 5 mm clips. Trocar site hernia (TSH) is a rare complication following LC, occurring in fewer than 1% of cases [20]. However, it is most often observed at sites where a 10 mm trocar was used [20]. Undoubtedly, trocar size is not the only risk factor for TSH; other factors, such as obesity and sex, have also been linked to this complication. Based on these findings, we believe that the routine use of a 10-12 mm epigastric trocar for LC is not recommended.

There are several limitations to our study. First, it is a retrospective analysis conducted at a single institution. As such, the decision to use an unusual CD closure method during surgery was made by the surgeons based on their clinical judgment. Second, we excluded patients who did not undergo MRCP before surgery, which may have resulted in a different cutoff value for CD size based on MRCP findings. Moreover, we assessed the length of the CD using MRCP images, which primarily represent the intraluminal capacity of the CD. MRCP primarily employs T2-weighted MRI pulse sequences, which produce high-intensity signals for fluids within the biliary and pancreatic ducts [21]. Consequently, the true CD diameter value may differ between those obtained via MRCP images and through intraoperative findings. We found that a CD diameter greater than 4.22 mm necessitated the use of 10 mm clips or an LS. However, some patients with a CD diameter below this threshold may still require 10 mm clips or alternative devices due to localized CD enlargement. Finally, we did not assess which device is the most effective among the unconventional closure methods. Our results showed that the use of suturing, a ligature device, and 10 mm clips was more common than an LS. Several researchers have reported the efficiency and safety of LS [12,13]. However, Thomas et al. noted that the use of LS may be associated with postoperative complications of Clavien-Dindo grade ≥3 [15]. Further, the comparison between 10 mm clips and LS has not been thoroughly investigated, leaving the question of which device is most effective unresolved. Further research is needed to clarify this issue.

## Conclusions

In this study, 20 (13%) patients who underwent LC required an alternative CD closure method such as a 10 mm clip, an LS, or a ligating device. This proportion of patients is not very high, but several young surgeons may have experienced this situation even if the cholecystitis had not been present. If a dilated CD has been clearly detected, dissection for it is not a difficult procedure in LC, but it is important to anticipate the CD closure method preoperatively. Our results showed that a dilated CD may contribute to prolonged operative time and increased intraoperative bleeding with or without cholecystitis. Therefore, preparing for unexpected bleeding or intraoperative complications would be required if the CD was observed to be enlarged via preoperative MRCP imaging. We identified the CD diameter cutoff value that could complicate intraoperative manipulation during LC. These findings may provide valuable information for ensuring the safe performance of LC.
